# Prevalence and factors associated with stunting and thinness among school-age children in Arba Minch Health and Demographic Surveillance Site, Southern Ethiopia

**DOI:** 10.1371/journal.pone.0206659

**Published:** 2018-11-02

**Authors:** Eshetu Zerihun Tariku, Getaneh Alemu Abebe, Zeleke Aschalew Melketsedik, Befikadu Tariku Gutema

**Affiliations:** 1 Department of Public Health, Arba Minch University, Arba Minch, Ethiopia; 2 Department of Medical Laboratory Science, Bahir Dar University, Bahir Dar, Ethiopia; 3 Department of Nursing, Arba Minch University, Arba Minch, Ethiopia; University of Ghana, GHANA

## Abstract

**Introduction:**

Despite consistent economic growth in the country, malnutrition remains one of the major public health problems in Ethiopia. The prevalence of malnutrition and its associated factors are well studied among under-five children. However, there is a paucity of evidence among older children in developing countries including Ethiopia. The aim of the study was to assess the prevalence of stunting and thinness and their associated factors among school-age children.

**Methods:**

A community-based cross-sectional study was conducted among randomly selected 389 school-age children in Arba Minch Health and Demographic Surveillance Site, Southern Ethiopia, during April and May 2017. Height for age and body mass index for age z scores were calculated using WHO Anthro Plus software as indicators of stunting and thinness respectively. A binary logistic regression model was used to assess the association between independent and outcome variables.

**Results:**

The prevalence of stunting and thinness were 41.9% (95% CI: 37–47) and 8.0% (95% CI: 5.4–10.8) respectively. The likelihood of stunting was significantly higher among children within the age group of 12–14 years old (AOR = 2.97, 95% CI: 1.78–4.95); children who were male (AOR = 1.94, 95% CI: 1.21–3.10); children living in households with medium wealth terciles (AOR = 2.90, 95%CI: 1.39–6.04); and children who were non-enrolled in schools (AOR = 2.25, 95% CI: (1.37–3.70). Moreover, thinness was 63% less common among children who had a dietary diversity score of <4 food groups (AOR = 0.37, 95%CI: 0.16–0.89).

**Conclusion:**

The prevalence of thinness is low when compared to the prevalence reported by a single national school health and nutrition survey in Ethiopia. Stunting is a major public health concern. Therefore, this finding warrants the need to implement school health and nutrition programs to improve the nutritional status of school-age children in the study area. The interventions should focus towards both gender and special emphasis should also be given to increase the enrollment of children in schools. In addition, effort should be taken by stakeholders in different hierarchies to improve the family wealth status.

## Introduction

School-age is a dynamic period of growth and development as children of this age group undergo rapid physical, mental and emotional development. Their social interaction also broadens out of their family, which poses an additional risk through reduced nutritional care and support. Like that of under-five children, school-age children are the most vulnerable segments of the population and seek special attention including their nutrition [1, 2].

Under-nutrition is widespread among school children (particularly in South Asia and Africa), and their nutritional status often worsens during their school years. It greatly affects both the cognitive and physical development of children [3, 4]. Thinness has been adopted recently as a more appropriate indicator of relatively recent nutritional deprivation, such as insufficient dietary intakes of energy, protein, or several micronutrients, than underweight in older children [5].

Globally, more than 200 million school-age children are stunted and underweight and if no action is taken, the number will grow to nearly a billion by 2020 [6–9]. Despite the economic growth observed in developing countries, under-nutrition is still highly prevalent [10]. Children in low and middle-income countries have been known to be at increased risk of under-nutrition due to poverty and lack of food [11]. Children’s physiology, the influence of the family and the community on their behavior may play an important role in the proper development and nutritional status of the child [12–14].

In Ethiopia, child malnutrition continues to be a major public health problem. According to the Ethiopian Demographic Health and Survey (EDHS) of 2016, the prevalence of wasting, underweight and stunting in children under 5 years is high (9.9%, 23.6%, and 38.4% respectively) [15]; while the situation in older children is not well studied [16,17]. The National School Health and Nutrition (SHN) survey conducted in 2008 showed that nearly 23% of children were stunted and a similar percentage of them were also thin for age [18].

Malnutrition among children has lifelong implications; its outcomes not only cover the whole life but are also transferred from one generation to another. Health problems due to poor nutritional status in school-age children are among the most common causes of low school enrolment, high absenteeism, early dropout, unsatisfactory classroom performance, delayed cognitive development, short stature, reduced work capacity, and poor reproductive performance [19–22].

Despite advocacy for health and nutrition services in primary schools, there is a clear paucity of information on the actual nutritional status of children in this age group in developing countries. Most studies focus on malnutrition in pregnant mothers and young children less than 5 years of age, whereas school-age children are often omitted from health and nutrition surveys or surveillance [23]. In Ethiopia, routine surveillance for under-nutrition among school-age children has not been conducted and there are limited data on school-age children about the prevalence and predisposing factors. Therefore, this study was designed to assess the prevalence of stunting and thinness and their associated factors among school-age children in Arba Minch Health and Demographic Surveillance Site (HDSS), Arba Minch Zuria district, Southern Ethiopia.

## Materials and methods

### Study design and setting

Community-based cross-sectional study with a quantitative method of data collection was conducted during April and May 2017 among school-age children (6 to 14 years old) in Arba Minch HDSS, Southern Ethiopia. Arba Minch Zuria district has 31 kebeles (the smallest administrative unit of Ethiopia) among which 9 kebeles are used as a target of Arba Minch HDSS which is being run by Arba Minch University. The administrative office of the district is in Arba Minch Town, which is located about 454 KMs south of Addis Ababa. According to the HDSS report, 74, 157 populations are living in the surveillance site, of whom 20,062 (27.1%) are aged between 6–14 years.

### Sample size and sampling technique

The sample size was calculated using single population proportion formula through the assumptions of 95% Confidence Interval (CI), 5% margin of error, and 39.8% prevalence of stunting among school-age children from a research conducted in Libo Kemkem and Fogera districts, Northwest Ethiopia [24]. Finally, considering 10% for non-response, the total sample size was 405. The source population was all school-age children (between 6 to 14 years old) residing in nine kebeles of Arba Minch HDSS. Children residing in the study area for > 6 months were included while the exclusion criteria considered were children who were critically ill during the time of data collection and those with physical deformities for anthropometric measurements. For the selection of the study participants, a dataset created which contains the list of an individual in the nine kebeles with their date of birth, individual and household identification using Arba Minch HDSS database. Using this dataset sampling frame was generated for children age from 6–14 years old. Study participants were randomly selected from each kebeles. The proportion of sample was allocated based on the number of school-age children in each kebeles.

### Data collection tools and procedures

Data was collected using structured, pretested Amharic version questionnaires. The study instruments included two sets of structured questionnaires. One was administered for children and another for the child’s mothers/caregivers. The data on household wealth index was collected by asking ownership of selected assets based on EDHS 2011 wealth index variables [25]. Household food insecurity level was measured with Household Food Insecurity Access Scale (HFIAS), which was developed and validated by Food and Nutrition Technical Assistant (FANTA) [26]. Child dietary intake was measured by qualitative 24 hour dietary recall of the child [27]. Children were interviewed with the presence of guardian.

#### Anthropometric measurements

Weight and height of each child were measured following standard anthropometric techniques [28]. For weight measurement, children were asked to remove their shoes, wear light clothes and remove accessories. Then, trained data collectors measured children’s weight on a calibrated portable digital scale (Seca, Germany model) and record the value to the nearest 0.1 kilograms. For height measurement, children were asked to stand erect with their shoulders level, hands at their sides, thighs and heels comfortably together, the buttocks, scapulae, and head are positioned in contact with the vertical backboard with a sliding head bar. Then height values were recorded to the nearest 0.1 centimeters [28].

### Data quality control

A pretest was done prior to actual data collection by recruiting 20 children and their guardians in one of HDSS kebele on households which were not selected. The final version of the questionnaire was translated into Amharic language and again translated back to English to check the consistency. The data collectors were given two days of intensive training on the instruments, method of data collection, how to take anthropometric measurements, ethical issues and the purpose of the study. Data collectors’ accuracy of anthropometric measurements was standardized with their trainer during training and pretesting. During data collection, data collectors have taken two separate height and weight measurements for an individual and average value were reported. The functionality of digital weight scales was checked using known weight every morning before data collection begins and before every weight measurement. Data collectors ensured the scale reading exactly at zero [28]. Investigators have checked the collected data for completeness and consistency throughout the data collection period.

### Data analysis

Data were entered into Epi Data version 3.1 and cleaned for inconsistency. For further analysis, the data were exported to Statistical Package for Social Science (SPSS) version 22 software. Descriptive analysis of data was indicated using numerical summary measures. Outliers and influential cases were also checked. The wealth index was constructed via Principal Component Analysis (PCA) method [29]. For anthropometric data analysis, standard deviation (Z-scores) scores were obtained by WHO Anthro Plus software to determine the nutritional status of children. Children whose Height for Age z-score (HAZ) and BMI for Age z-score (BAZ) above-2SD scores were considered as well-nourished and those below -2SD scores as being malnourished (stunted and thin respectively). The collinearity effect was checked using variance inflation factor (VIF) and standard error and non-collinear covariates were included in the independent binary logistic regression model to assess the possible association of independent and outcome variables. Independent variables with p-value ≤ 0.25 in bivariate analysis were included for multivariate analysis [30]. Additionally, context and previous studies were also considered to make a variables candidate for multivariate analysis. Statistical significance at p-value < 0.05 in multivariable analysis was considered. The fitness of the model was tested by Hosmer- Lemeshow goodness of fit test.

### Ethical considerations

Ethical approval was obtained from Institutional Ethics Review Board (IRB) of Arba Minch University. Official permission letter was obtained from Arba Minch Zuria district administrative and health offices and the data collection begun after permission and cooperation letter was written to all nine kebeles where the study was carried out. Individual informed written consent and assent was obtained and the respondents were assured of confidentiality.

## Results

### Socio- economic and demographic characteristics of study participants

A total of 389 school-age children with their mothers/caregivers were participated in the study making a response rate of 96%. The mean ±SD age of children was 10.14±2.6 years. Among the total children participated, 222 (57.1%) were enrolled in school. Two hundred seventy-one (69.7%) of interviewed caregivers were females and the majority (65.6%) of mothers/ caregivers did not attend formal education. From participated households, 183 (47.0%) of households were food secured (Table 1).

**Table 1:** Socio-demographic and economic characteristics of study participants (n = 389), Arba Minch HDSS, Southern Ethiopia, April to May 2017.

Variables	Category	Frequency	Percentage
Age of child (in years)	6–11	252	64.8
	12–14	137	35.2
Child sex	Male	194	49.9
	Female	195	50.1
School Enrolment	Enrolled	222	57.1
	Non enrolled	167	42.9
Grade (n = 222)	1–4 class	169	76.1
	5–8 class	53	23.9
Child birth order	≤ 2 born into the family	199	51.2
	>2 born in to the family	190	48.8
Age of parent/ care giver	18–35	191	49.1
	36–45	145	37.3
	>45	53	13.6
Sex of parent/ care giver	Male	118	30.3
	Female	271	69.7
Religion of parent/ care giver	Orthodox	141	36.2
	Protestant	240	61.7
	Others	8	2.1
Educational status of mothers/care takers	No formal education	256	65.8
	Unable to read and write	38	9.8
	Primary level	82	21.1
	Secondary level and above	13	3.3
Educational status of father	No formal education	215	55.3
	Unable to read and write	43	11.1
	Primary level	106	27.2
	Secondary level and above	25	6.4
Occupation of mother/ care giver	Housewife	369	94.9
	Private employee	14	3.6
	Others	6	1.5
Occupation of father	Government employee	11	2.8
	Private employee	217	55.8
	Farmer	133	34.2
	Daily laborer	18	4.6
	Merchant	8	2.1
	Others	2	0.5
Family size	<4	18	4.6
	≥4	371	95.4
Family wealth terciles	Poor	129	33.2
	Medium	131	33.7
	Rich	129	33.2
Household food security status	Food secured	183	47.0
	Food in-secured	206	53.0

### Child health, dieting habit and environmental characteristics

Only 16 (4.1%) children experienced illness two weeks preceding data collection period. One hundred eighty-five (47.6%) children had the habit of missing any of meal schedule. More than half of the children (60.9%) had a Dietary Diversity Score (DDS) of ≥ 4 groups. Nearly all surveyed households (95.6%) had latrine and the most common (77.4%) source of drinking water was pipe water (Table 2).

**Table 2:** Child health, dieting habit and environmental characteristics of study participants (n = 389), Arba Minch HDSS, Southern Ethiopia, April to May 2017.

Variables	Category	Frequency	Percentage
Illness/infection in the last two weeks prior to the survey	Yes	16	4.1
	No	373	95.9
Habit of hand washing before going to meal	Yes	275	70.7
	No	114	29.3
Habit of missing some meal schedules	Yes	185	47.6
	No	204	52.4
Child DDS	<4 groups	152	39.1
	≥ 4 groups	237	60.9
Latrine availability at home	Yes	372	95.6
	No	17	4.4
Waste disposal system	Pit	152	39.1
	Burning	105	27.0
	Garbage can	54	13.9
	Open field	78	20.0
Source of drinking water	Pipe water	301	77.4
	Protected well/ spring	36	9.3
	Unprotected well/ spring	37	9.5
	River	15	3.9
Mothers/care-givers ever received health or nutrition related information regarding child care	Yes	182	46.8
	No	207	53.2

### Nutritional status of school-age children (N = 389)

The mean (SD) HAZ and BAZ of school-age children were -1.92 (2.02) and -0.15 (1.81), respectively. Using WHO growth reference for school- age children [31], 41.9% (95% CI: 37–47) were stunted and 8.0% (95% CI: 5.4–10.8) were thin. The prevalence of severe form of stunting (HAZ <-3SD) and thinness (BAZ < -3SD) among school-age children were 26.0% (95% CI: 21.9–30.8) and 3.6% (95%CI: 2.1–5.4) respectively.

### Factors associated with stunting and thinness among school-age children

In the multivariable logistic regression analysis, the likelihood of stunting was significantly higher among children within the age group of 12–14 years old (AOR = 2.97, 95% CI: 1.78–4.95); male (AOR = 1.94, 95% CI: 1.21–3.10); those who live in households with medium family wealth terciles (AOR = 2.90, 95%CI: 1.39–6.04); and non-enrolled in schools (AOR = 2.25, 95% CI: (1.37–3.70). Children who had a habit of missing some meal schedules (AOR = 0.43, 95% CI: 0.26–0.72) were associated with decreased odds of stunting. Moreover, thinness was 63% less common among children who had DDS of <4 food groups than their counterparts who had DDS of ≥4 food groups (AOR = 0.37, 95%CI: 0.16–0.89) (Tables 3 and 4).

**Table 3:** Factors associated with stunting among school-age children (n = 389), Arba Minch HDSS, Sothern Ethiopia, 2017.

Covariates	Category	Stunted (HAZ < -2SD)		COR (95%CI)	AOR (95%CI)
		Yes. N (%)	No. N (%)		
Age of child (in years)	6–11	93 (36.9)	159(63.1)	1	1
	12–14	70 (51.1)	67 (48.9)	1.79(1.17–2.72)	2.97 (1.78–4.95)*
Child sex	Male	92 (47.4)	102(52.6)	1.58(1.05–2.36)	1.94 (1.21–3.10)*
	Female	71 (36.4)	124(63.6)	1	1
School Enrolment	Enrolled	76 (34.2)	146 (65.8)	1	1
	Non enrolled	87 (52.1)	80 (47.9)	2.01(1.39–3.15)	2.25 (1.37–3.70)*
Sex of parent/care giver	Male	67 (56.8)	51 (43.2)	2.40(1.54–3.72)	1.52 (0.91–2.54)
	Female	96 (35.4)	175 (64.6)	1	1s
Educational status of mother	No formal education	145 (49.3)	149 (50.7)	4.16 (2.37–7.30)	1.55 (0.74–3.24)
	Formal education	18 (18.9)	77 (81.1)	1	1
Educational status of father	No formal education	131 (50.8)	127 (49.2)	3.20 (2.00–5.09)	1.53 (0.83–2.81)
	Formal education	32 (24.4)	99 (75.6)	1	1
Family wealth terciles	Poor	65 (50.4)	64 (49.6)	3.66(2.13–6.30)	2.15 (1.00–4.60)
	Medium	70 (53.4)	61 (46.6)	4.14(2.41–7.11)	2.90 (1.39–6.04)*
	Rich	28 (21.7)	101 (78.3)	1	1
Household food security status	Food secure	77 (42.1)	106 (57.9)	1	1
	Food insecure	86 (41.7)	120 (58.3)	0.95(0.66–1.48)	0.88 (0.54–1.42)
Habit of missing some meal schedules	Yes	66 (35.7)	119 (64.3)	0.61(0.41–0.92)	0.43 (0.26–0.72)*
	No	97 (47.5)	107 (52.5)	1	1
Child DDS	<4 groups	83 (54.6)	69 (45.4)	2.36(1.56–3.58)	1.30 (0.76–2.21)
	≥4 groups	80 (33.8%)	157 (66.2)	1	1

Note: AOR = Adjusted Odd Ratio; CI = Confidence Interval, COR = Crude Odd Ratio

* = p-value <0.05.

**Table 4:** Factors associated with thinness among school-age children (n = 391), Arba Minch HDSS, Southern Ethiopia, 2017.

Variables	Category	Thinness (BAZ<-2SD)		COR (95%CI)	AOR (95%CI)
		Yes N (%)	No N (%)		
Age of child (in years)	6–11	17 (6.7)	235 (93.3)	0.64(0.30–1.33)	0.60 (0.28–1.27)
	12–14	14 (10.2)	123 (89.8)	1	1
Family size	<4	3 (16.7)	15 (83.3)	1	1
	> = 4	28 (7.5)	343 (92.5)	0.41(0.11–1.50)	0.52 (0.14–2.01)
Average estimated monthly income	<500	26 (9.1)	261 (90.9)	4.58(0.61–34.60)	5.00 (0.63–39.60)
	500–999	4 (7.3)	51 (92.7)	3.61(0.39–33.46)	3.77 (0.40–35.44)
	> = 1000	1 (2.1)	46 (97.9)	1	1
Household food security status	Food secure	9 (4.9)	174 (95.1)	1	1
	Food insecure	22 (10.7)	184 (89.3)	2.31(1.04–5.16)	2.13 (0.91–4.98)
Child DDS	<4 groups	8 (5.3)	144 (94.7)	0.52(0.23–1.19)	0.37 (0.16–0.89)*
	≥ 4 groups	23 (9.7)	214 (90.3)	1	1

Note: AOR = Adjusted Odd Ratio; CI = Confidence Interval, COR = Crude Odd Ratio;

* = p-value <0.05.

## Discussion

In the present study, the prevalence of stunting was found to be 41.9% which is comparable with the study from Libo Kemkem and Fogera, Ethiopia (39.8%) and Obafemi Owode, Nigeria (40.3%) [24, 32]. However, the prevalence in this study is lower than the prevalence reported from Nkwanta district, Ghana (50.3%) [33]. The difference could be attributed to the difference in the study period, variation in the age category of target populations and the recently initiated nutrition sensitive intervention activities in the study area.

When compared with the result from China (11.68%) [34], India (18.5%), and Mexico (22.3%) [35], the prevalence of stunting in this study was much higher. The prevalence we found in this study was also higher than the findings reported from different parts of Ethiopia; Gondar (23%) [36], Fogera (30.7%) [8] and Somali (32.96%) [37]. It is also much higher than the result from Kersa and Addis Ababa where the prevalence of stunting is below 20% [38, 39]. Again it is higher than the prevalence of stunting report by national SHN survey, Ethiopia (23%) [40]. From nine kebeles of the study site of Arba Minch HDSS, one is semi-urban, the remaining are rural. Higher prevalence of stunting in this study could be due to the difference in residency characteristics of the study subjects.

In this study, about 8.0% of school-age children living in Arba Minch HDSS were thin. This finding is nearly similar to the report from Pakistan where the prevalence of thinness was 10% [41]. Nonetheless, this prevalence is lower than the finding reported from Ghana (19.4%) [33] and the prevalence reported by the study from Fogera, Ethiopia (21.4%) [24]. It is also lower than the finding from the national SHN survey, Ethiopia (23%) [40]. The decreased prevalence of thinness in this study might be related with season variation of the study period. In the study area, April and May are among the major crop production periods (wet season) which may contribute to the decreased prevalence of thinness. This is supported by previous studies in Ethiopia where seasonal variations in the prevalence of under-nutrition have been reported [42, 43]. In addition, the presence of relatively minimum percent (4.1%) of children affected by illness, one of the major causes of acute malnutrition, in this study might be contributed to the decreased prevalence.

Poor nutrition arises from multifaceted and interrelated circumstances and determinants, from immediate to underlying causes. The present study showed that the age of children was significantly associated with stunting. Older children were more likely to be stunted than the younger age group. A study in Northern, Ethiopia [24] and India [44] among school-age children showed a similar relationship between age and stunting, especially for children from 5–14 years old. This pattern might be an indication for the continuity of the process of stunting during school age in low and middle-income countries. This could probably be due to older children are in the transition life stage to adolescence when several unique challenges, including an increased body requirement for nutritional need, are observed [45].

Various studies showed that under-nutrition exists in both sexes and in different age groups differently [6, 44, 46]. In the present study, stunting was common among males than females. This is in agreement with those of other studies among school-age children in Ethiopia [24], India [6] and Sir Lanka [46]. This could be because males’ growth and development is more influenced by environmental and nutritional stress (including common childhood illnesses) than females and thus, making males more likely to be affected by chronic under-nutrition [47].

This study revealed that the odds of being stunted were more common among children who were non-enrolled in school than enrolled children. This could probably result from the possibility that non-enrollment in schools increased children’s participation in work for pay outside the household which may consequently attribute to an imbalance in their body’s nutritional intake and requirements [48]. This may have an important implication that school-based health and nutrition interventions programs should be planned in a way that will also benefit non-enrolled children.

The economic status of a household where a child lives has been identified as one of the key determinants of child nutritional status and it is stated that household economic status significantly affects access to food [49]. In this study, children in lower wealthy households were significantly associated with increased risk of stunting than children in wealthier households. The odds of stunting were three times more among children from medium family wealth terciles than children from rich family wealth terciles. These findings concurred with the result from previous research in Pakistan [41], and provide further evidence that household wealth inequality is an important risk factor for child chronic under-nutrition.

Inappropriate feeding practices could have a negative effect on child growth and development, especially in developing countries, where the accessibility of basic health service is not sufficient [6, 50]. The present study, however, affirmed that the likelihood of stunting was 57% less likely among children who had a habit of missing some meal schedules than those who did not miss their meal schedules. This might be explained by stunting is a chronic form of under-nutrition and the short term dietary habit of the child would have a minimal contribution.

In this study, the covariate, child DDS was found to be negatively associated with thinness among school-age children. Children with DDS of <4 groups were 63% less likely to be thin than those children with DDS of ≥ 4 groups. This could, however, be an area where further research is needed.

Being community-based, this study provided evidence which could be representative of all school-age children, in Arba Minch HDSS. This could be very essential for planning intervention strategies which will equally benefit school-age children regardless of their enrollment status in the schools.

One of the major limitations of this study was since the study was conducted in a wet season; it might underestimate the prevalence of thinness (acute under-nutrition). Second, a cross-sectional design was used and it was difficult to establish the cause-effect relationship between the outcome variable and the covariates. However, since there is supporting evidence, the effect estimated in this study could be a good measure of associations between the identified factors and child under-nutrition.

## Conclusion

In this study, the prevalence of thinness is low when compared to the prevalence reported by a single national school health and nutrition survey in Ethiopia. However, the prevalence of stunting is very high and it is considered as a major public health concern among school-aged children in Arba Minch HDSS, Southern Ethiopia. The likelihood of stunting was significantly higher among older school age children and children who were non-enrolled in schools. Being male, and living in households with lower wealth index were also associated with increased odds of stunting. Therefore, this finding warrants the need to implement school health and nutrition programs to improve the nutritional status of school-age children in the study area. The interventions should focus towards both gender and special emphasis should also be given to increase the enrollment of children in schools. In addition, effort should be taken by stakeholders in different hierarchies to improve the family wealth status. Moreover, more-large scale studies or the national nutritional surveys need to consider school-age children as one component to regularly assess the nutritional status of this age group.

## Supporting information

S1 Tool This is the S1 English version tool. (DOCX)

S2 Tool This is the S2 Amharic version tool. (DOCX)

S1 Data This is the S1 SPSS data set. (SAV)
