# Supplementary material for: Prevalence and factors associated with stunting and thinness among school-age children in Arba Minch Health and Demographic Surveillance Site, Southern Ethiopia
Source: PLoS One. 2018 Nov 2;13(11):e0206659. doi: 10.1371/journal.pone.0206659 (PMC6214544; doi:10.1371/journal.pone.0206659)
Supplement: S1 Tool — (DOCX) [file pone.0206659.s001.docx]

## English Version Questionnaire

**Part I: Questionnaire for the child**

Participant code ________ Households code_______ kebele ------------- Date**----------------**

| **Socio-demographic Characteristics** | |  | |
| --- | --- | --- | --- |
| **Question** | | **Response** | **skip rule** |
| 101. | Age | **_______________** |  |
| 102. | Sex | 1. Male  2. Female |  |
| 103 | Are you enrolled to school | - - - 1. Yes       2. No | If no go to Q 105 |
| 104 | If enrolled, grade you are attending | __________ |  |
| 105 | What is your birth order? (Which child are you in your family?) | __________ |  |
| 106 | With whom you are living with (more than one answer is possible) | 1. Mother  2. Father  3. Brother or sister  4. Others (specify------------) |  |
| 107 | Have you told by health professional as you experienced any illness/ infection in the last 2 weeks? | 1. yes  2. No | If no go to Q 109 |
| 108 | If yes to Q 107, what type of illness did you experienced? | 1. Diarrhea  2. malaria  3. parasitic infection  4. Others (specify)________ |  |
| 109 | Do you have habit of washing your hand before going to meal? | 1. yes  2. no |  |

| **Dieting habit** | | | | |
| --- | --- | --- | --- | --- |
| The next questions ask about fruits and vegetables that you usually eat. | | | | |
|  | | **Question** | **Response** | **skip rule** |
| 1. . | In a typical week on how many days do you eat fruit? | | **____________** | If zero go to Q 203 |
| 1. . | How many **serving** of fruit do you eat on one of those days? | | ________________ |  |
| 1. . | In a typical week on how many days do you eat vegetables? | | **____________________** | If zero go to Q 205 |
| 1. . | How many servings of vegetables do you eat on one of those days? | | __________________ |  |
|  | In a typical week on how many days do you eat meat/meat products | | ______________________ | If zero go to Q 207 |
|  | How many servings of meat do you eat on one of those days? | | ______________________ |  |
| 207. | How do you regularly get your lunch? | | 1. Bring from home 2. Buy from school cafeteria 3. Buy from nearby food service establishment. 4. I did not use lunch |  |
| 208. | In the last two weeks, do you have a regular habit of missing any of your meal schedules? | | 1. Yes 2. No |  |

**Questions to assess child dietary consumption pattern**

**Instruction:** Ask the child to recalls all the foods and beverages consumed yesterday during the day and night, whether at home or outside the home., underline the corresponding foods in the list under the appropriate food group and write “1” in the column next to the food group if at least one food in this group has been underlined. Once the recall is finished, probe for food groups where no food was underlined.

| **Q. No** | **Food group** | **Response Yes (1) No (0)** |
| --- | --- | --- |
| 301 | Injera, kita, qollo, pourage, bread, rice, biscuits, or any other foods made from millet, barely, oat, sorghum, maize, rice, wheat, or teff |  |
| 302 | Potatoes, yams, manioc, cassava or any other foods made from roots or tubers |  |
| 303 | Vitamin A rich vegetables and tubers like Pumpkin, carrot, or sweet potato, red sweet pepper |  |
| 304 | Dark green leafy vegetables like cabbage, lettuce, spinach? |  |
| 305 | Other fruits and vegetables like tomato, onion, lemon |  |
| 306 | Any vitamin A rich fruits like avocado, mango, papaya or banana and 100% fruit juice made from these |  |
| 307 | Liver, kidney, heart or other organ meats or blood-based foods |  |
| 308 | Any beef, lamb, goat, chicken, or other birds, liver |  |
| 309 | Any eggs |  |
| 310 | Any fresh or dried fish or shellfish |  |
| 311 | Any foods made from beans, peas, lentils, or nuts |  |
| 312 | Any cheese, yogurt, milk or other milk products |  |
| 313 | Any foods made with oil, fat, or butter |  |
| 314 | Any sugar or honey |  |
| 315 | Any other foods, such as condiments, coffee, tea, local bear, alcohol? |  |

**Child physical measurements (to be filled by data collectors)**

| **Measurements** | | **Response (average value)** | **Skip rule** |
| --- | --- | --- | --- |
| 401. | Height | In centimeters **_____________** |  |
| 402. | Weight | In kilograms (Kg) ________ |  |

**Part II: Questionnaire for the child family**

Participant code__________ Date **____________**

| S. No | **Demographic information** | **Response** | **skip rule** |
| --- | --- | --- | --- |
| 501 | Respondent age | **_____________________** |  |
| 502 | Respondent sex | 1. Male 2. Female |  |
| 503 | Religion of parents | 1. Muslim 2. Orthodox 3. Catholic 4. Protestant 5. Others (specify)_________ |  |
| 504 | Occupation of the Mothers/care taker? | 1. House wife 2. Government Employee 3. Private Business 4. Others (specify)_________ |  |
| 505 | Occupation of father | 1. Government Employee 2. Private Business 3. Farmer 4. Daily laborer 5. Merchant 6. Others (specify)………… |  |
| 506 | Ethnicity | 1. Gamo 2. Gofa 3. Oromo 4. Amhara 5. Tigre 6. Others (specify)__________ |  |
| 507 | Maternal education | 1. No formal education 2. Doesn’t read and write 3. Primary education 4. Secondary education and above |  |
| 508 | Fathers education | 1. No formal education 2. Doesn’t read and write 3. Primary education 4. Secondary education and above |  |
| 509 | How many people including yourself, live in your household? | _________________ |  |
| 510 | What is the average estimated monthly earning of the family in birr? | **_________________** |  |
| 511 | Have you ever been diagnosed pregnancy related Diabetes Mellitus in any of your pregnancy? (only for female respondents) | 1. Yes 2. No |  |
| 512 | Do you have latrine in the compound/ household? | 1. yes 2. no |  |
| 513 | How you disposed the waste generated in the compound? | 1. no 2. pit 3. burning 4. open field 5. garbage can |  |
| 514 | Source of drinking water | 1. pipe water 2. protected well/ spring 3. unprotected well/ spring 4. river |  |
| 515 | Have you ever used bed net for your child having an interview know? | 1. yes 2. no |  |
| 516 | Have you ever received any health or nutrition related information regarding the child care? | 1. yes  2. no | If 2 go to Q 601 |
| 517 | If yes to Q 519, from where do you get the information? | 1. from health facilities  2. from HEW (outside health facility)  3. others (specify)_________ |  |

**Questions to assess household food security condition (HFIAS)**

| **Q. No** | **Questions** | **Response options (encircle one)** | | | **skip** |
| --- | --- | --- | --- | --- | --- |
| 601. | In the past four weeks, did you worry that your household would not have enough food? | | 0 = No  1=Yes | | If 0, Q 602 |
| 601.a | How often did this happen? | 1 = Rarely (1X or 2X in the past four weeks)  2 = Sometimes (3x to 10x in the past four weeks)  3 = Often (>10x in the past four weeks) | | |  |
| 602. | In the past four weeks, were you or any household member not able to eat the kinds of foods you preferred because of a lack of resources? | | 0 = No  1=Yes | | If 0, to Q 603 |
| 602.a | How often did this happen? | 1 = Rarely (1X or 2X in the past 4 weeks)  2 = Sometimes (3x to 10x in the past four weeks)  3 = Often (>10x in the past four weeks) | | |  |
| 603 | In the past four weeks, did you or any household member have to eat a limited variety of foods due to a lack of resources? | | 0 = No  1=Yes | | If 0, to Q 604 |
| 603.a | How often did this happen? | 1 = Rarely (1X or 2X in the past four weeks)  2 = Sometimes (3x to 10x in the past four weeks)  3 = Often (>10x in the past four weeks) | | |  |
| 604 | In the past four weeks, did you or any household member have to eat some foods that you really did not want to eat because of a lack of resources to obtain other types of food? | | 0 = No  1=Yes | | If 0, to Q 605 |
| 604.a | How often did this happen? | 1 = Rarely (1X or 2X in the past four weeks)  2 = Sometimes (3x to 10x in the past four weeks)  3 = Often (>10x in the past four weeks) | | |  |
| 605 | In the past four weeks, did you or any household member have to eat a smaller meal than you felt you needed because there was not enough food? | | 0 = No  1=Yes | | If 0, to Q 606 |
| 605.a | How often did this happen? | 1 = Rarely (1X or 2X in the past four weeks)  2 = Sometimes (3x to 10x in the past four weeks)  3 = Often (>10x in the past four weeks) | | |  |
| 606 | In the past four weeks, did you or any other household member have to eat fewer meals in a day because there was not enough food? | | 0 = No  1=Yes | | If 0, to Q 607 |
| 606.a | How often did this happen? | 1 = Rarely (1X or 2X in the past four weeks)  2 = Sometimes (3x to 10x in the past four weeks)  3 = Often (>10x in the past four weeks) | | |  |
| 607 | In the past four weeks, was there ever no food to eat of any kind in your household because of lack of resources to get food? | | 0 = No  1=Yes | | If 0, to Q 608 |
| 607.a | How often did this happen? | 1 = Rarely (1X or 2X in the past four weeks)  2 = Sometimes (3x to 10x in the past four weeks)  3 = Often (>10x in the past four weeks) | | |  |
| 608 | In the past four weeks, did you or any household member go to sleep at night hungry because there was not enough food? | | 0 = No  1=Yes | | If 0, to Q 609 |
| 608.a | How often did this happen? | 1 = Rarely (1X or 2X in the past four weeks)  2 = Sometimes (3x to 10x in the past four weeks)  3 = Often (>10x in the past four weeks) | | |  |
| 609 | In the past four weeks, did you or any household member go a whole day and night without eating anything because there was not enough food | | | 0 = No (Questionnaire finished)  1=Yes | If 0, to next part |
| 609.a | How often did this happen? | 1 = Rarely (1X or 2X in the past four weeks)  2 = Sometimes (3x to 10x in the past four weeks)  3 = Often (>10x in the past four weeks) | | |  |

**Questions to assess the current family wealth (economic condition)**

Could you tell me if you have the following in your house?

| **Asset type** | **Response** | |
| --- | --- | --- |
| **Domestic animals** |  |  |
| Ox | No (0) | Yes (1) |
| Cow | No (0) | Yes (1) |
| Calf | No (0) | Yes (1) |
| Sheep | No (0) | Yes (1) |
| Goat | No (0) | Yes (1) |
| Horse | No (0) | Yes (1) |
| Donkey | No (0) | Yes (1) |
| Cock and Hen | No (0) | Yes (1) |
| **Durable assets** |  |  |
| Television | No (0) | Yes (1) |
| Radio | No (0) | Yes (1) |
| Electricity | No (0) | Yes (1) |
| Refrigerator | No (0) | Yes (1) |
| Conventional telephone | No (0) | Yes (1) |
| Mobile phone | No (0) | Yes (1) |
| Car | No (0) | Yes (1) |
| Motorcycle | No (0) | Yes (1) |
| Cycle | No (0) | Yes (1) |
| Cart | No (0) | Yes (1) |
| Gold, money | No (0) | Yes (1) |
| Ownership of owned living house | No (0) | Yes (1) |
| Ownership of agricultural land | No (0) | Yes (1) |
| **Productive assets** |  |  |
| Plough plow | No (0) | Yes (1) |
| Axe | No (0) | Yes (1) |
| Hoe | No (0) | Yes (1) |
| Shovel | No (0) | Yes (1) |
| Sickle | No (0) | Yes (1) |
| Beehive | No (0) | Yes (1) |
| **Housing characteristics** |  |  |
| Indoor plumping/ pipe water | No (0) | Yes (1) |
| Type of flooring | Earth/dung (0) | Cement/raw wood (1) |
| Bed | No (0) | Yes (1) |
| Table | No (0) | Yes (1) |
| Chair | No (0) | Yes (1) |
| Stove | No (0) | Yes (1) |

END, Thank you for your participation!
