# Supplementary material for: Prevalence and factors associated with stunting and thinness among school-age children in Arba Minch Health and Demographic Surveillance Site, Southern Ethiopia
Source: PLoS One. 2018 Nov 2;13(11):e0206659. doi: 10.1371/journal.pone.0206659 (PMC6214544; doi:10.1371/journal.pone.0206659)
Supplement: S2 Tool — (DOCX) [file pone.0206659.s002.docx]

## መጠይቅ

**ክፍል አንድ: - ለልጁ/ ህጻኑ የሚጠየቅ መጠይቅ**

የተሳታፊው ኮድ__________ የመኖሪያ ቤት ኮድ.__________ ቀበሌ_________ ቀን………

| መሰረታዊ መረጃ | |  | |
| --- | --- | --- | --- |
| ጥያቄ | | ምላሽ | *ይለፉ* |
|  | ዕድሜ ስንት ነዉ? |  |  |
|  | ፆታ | 1. ወንድ 2. ሴት |  |
|  | መደበኛ ትምህርት ትማራለህ? | 1. አዎ 2. አልማርም | ‘2’ ከሆነ *ወደ ጥያቄ ቁጥር* 105 *ይለፉ* |
|  | መደበኛ ትምህርት የምትማር ከሆነ ስንተኛ ክፍል ነህ? |  |  |
|  | ስንተኛ ልጅ ነህ? | ____________________ |  |
|  | ከማን ጋር ነው የምትኖረው? | 1. እናት 2. አባት 3. ወንድም/እህት 4. ሌላ ከሆነ ጥቀስ________ |  |
|  | በጤና ባለሙያ ከሁለት ሳምንት በፊት ህመም እንደነበረብህ ወይም ታመህ እንደነበረ ተነግሮህ ነበር? | 1. አዎ 2. አልታመምኩም | 2’ ከሆነ *ወደ ጥያቄ ቁጥር* 109 *ይለፉ* |
|  | አዎ ካለክ በምን አይነት በሽታ ነበር የታመምከው? | 1. ማስቀመጥ 2. ወባ 3. የሆድ ትላትል 4. ሌላ ከሆነ ጥቀስ_______ |  |
|  | ምግብ ከመመገብህ በፊት እጅህን የመታጠብ ልምድ አለህ? | 1. አዎ 2. የለኝም |  |

| የምግብ አመጋገብ ልምድ | | | |
| --- | --- | --- | --- |
| ከዚህ ቀጥሎ ያሉት ጥያቄዎች የተለመደ የአትክልት እና ፍራፍሬ አመጋገብን ይመለከታል፡ | | | |
| ተ.ቁ | ጥያቄ | ምላሽ | እለፍ |
|  | በሳምንት ውስጥ ምን ያህል ቀናቶችን ፍራፍሬ ትመገባለህ? |  | ‘0’ ከሆነ ወደ ጥያቄ ቁጥር 203 እለፍ |
|  | በቀን ውስጥ ስንት ጊዜ ፍራፍሬ ትመገባለህ? |  |  |
|  | በሳምንት ውስጥ ምን ያህል ቀናቶችን አትክልት ትመገባለህ? |  | ‘0’ ከሆነ ወደ ጥያቄ ቁጥር 205 እለፍ |
|  | በቀን ውስጥ ስንት ጊዜ አትክልት ትመገባለህ? |  |  |
|  | በሳምንት ውስጥ ምን ያህል ቀናቶችን ስጋና የስጋ ወጤቶችን ትመገባለህ? |  | ‘0’ ከሆነ ወደ ጥያቄ ቁጥር 207 እለፍ |
|  | በቀን ውስጥ ስንት ጊዜ ስጋ ትመገባለህ? |  |  |
|  | የምሳ ምግብ ከየት ነው የምታገኘው/የምትበላው? | 1. ከቤት አመጣለሁ  2. ከት/ት ቤት ካፌ እገዛለሁ  3. በአቅራቢያ ካለ ምግብ ቤት እገዛለሁ  4. ምሳ አልበላም |  |
|  | ባለፈዉ 2 ሳምንት፣ ምግብ የመመገቢያ ሰዓቶችህን የመዝለል/የማሳለፍ ልምድ አለህ? | 1. አዎ  2. የለኝም |  |

**የህፃኑን/ ልጁን የምግብ አመጋገብ ሁኔታ የሚዳስስ መጠይቅ**

መመሪያ፤ - ህፃኑ/ልጁ በትናነትናዉ እለት ቀንና ማታ እንዲሁም ቤት ወይም ከቤት ውጪ የተጠቀማቸውን ምግብና መጠጦች እንዲያስታውስ እና እነዲናገር በመጠየቅ ልጁ የጠቀሳቸውን የምግብ አይነቶች ከታች በሰንጠረዡ ከተዘረዘሩት የመግብ ምድቦች በማየት ምልክት ወይም መስመር አድርግ/ጊ፡፡ ከምግብ ምድቦቹ ውስጥ በትንሹ አንድ የምግብ ዓይነት ከጠቀሰ ምላሽ በሚለው ላይ 1 ቁጥርን ጻፍ/ፊ፡፡

| ተ.ቁ | የምግቦች ምድቦች | ምላሽ   1. አዎ 2. የለም |
| --- | --- | --- |
|  | እንጀራ፣ ቂጣ፣ ቆሎ፣ አጥሚት፣ ዳቦ፣ እሩዝ፣ ብስኩት፣ ወይም ሌሎች ምግቦች ከ ዳጉሳ፣ ገብስ፣ ጤፍ፣ በቆሎ፣ አጃ፣ ማሽላ፣ ሩዝ፣ ስንዴ የተዘጋጁ |  |
|  | ድንች፣ የስር ተክል(ቦይና፣ ጎደሬ)፣ ካሳቫ ወይም ሌሎች የሚበሉ የስር ተክሎች |  |
|  | በቫየታሚን ኤ የበለጸጉ አትክልቶች እና ስራስሮች ማለትም ካሮት፣ ስካር ድንች፣ ቃሪያ |  |
|  | አረንጉዴ ቅጠላማ አትክልቶች እንደ ጎመን፣ ሰላጣ፣ ስፒናች ጎመን |  |
|  | ሌሎች ፍራፍሬዎች እና አትክልቶች እንደ ቲማቲም፣ ሽንኩርት፣ ሎሚ |  |
|  | በቫየታሚን ኤ የበለጸጉ ፍራፍሬዎች እነ አቦካዶ፣ ማንጎ፣ ፓፓያ፣ ወይም ሙዝ እና ከነዚህ የተሰራ 100 ጭማቂ |  |
|  | ጉበት፣ ኩላሊት፣ ልብ፣ ወይም ሌሎች የስጋ ክፍሎች/ደም የነካቸው ምግቦች |  |
|  | ማንኛውም የበሬ ስጋ፣ የበግ ጠቦት፣ የፍየል ስጋ፣ የዶሮ ስጋ፣ ወይም ሌሎች |  |
|  | ማንኛውም እንቁላል |  |
|  | ማንኛውም አዲስ ወይም የደረቀ ዓሳ |  |
|  | ከ ባቄላ፣ ሽንብራ፣ ምስር፣ ለወውዝ የተሰራ ማንኛውም ምግብ |  |
|  | ዓይብ፣ እርጎ፣ ወተት እና የወተት ተዕዋጽኦ |  |
|  | በዘይት፣፣ ስብ፣ ወይም ቅቤ የተሰራ ማንኛውም ምግብ |  |
|  | ማንኛውም ስካር ያለው ምግብ ወይም ማር |  |
|  | ሌሎች ምግቦች ለምሳሌ ቅመም-፣ ቡና፣ ሻይ፣ ቢራ፣ |  |

**የህፃናት የሰውነት ልኬቶች (በመረጃ ሰብሳቢው የሚሞላ)**

| የልኬት አይነት | | የልኬት ውጤት (አቭሬጅ) | እለፍ |
| --- | --- | --- | --- |
| 401. | ቁመት | በሴንቲሜትር= |  |
| 402. | ክብደት | በኪሎ ግራም (ኪግ)= |  |

**ክፍል ሁለት፡ ለህፃናት ወላጆች/አሳዳጊዎች የሚጠየቁ**

| ተ. ቁ. | መሰረታዊ መረጃ | ምላሽ | እለፍ |
| --- | --- | --- | --- |
| 501 | እድሜ |  |  |
| 502 | ፆታ | 1. 1. ወንድ 2. 2. ሴት |  |
| 503 | የወላጆች/ያሳዳጊዎች ኃይማኖት | 1. 1. ሙስሊም 2. 2. ኦርቶዶክስ 3. 3. ካቶሊክ 4. 4. ፕሮቴስታነት 5. 5. ሌላ (ይገለፅ)_________ |  |
| 504 | የእናት/ያሳዳጊ ተቀዳሚ ስራ? | 1. የቤት እመቤት 2. የመንግስት ሰራተኛ 3. የግል ስራ 4. ሌላ (ይገለፅ)_________ |  |
| 505 | የአባት ተቀዳሚ ስራ | ~~1.~~ የመንግስት ሰራተኛ   1. የግል ስራ 2. ገበሬ 3. የ ቀን ስራ 4. ነጋዴ 5. ሌላ (ይገለፅ)_________ |  |
| 506 | ብሔር | 1. ጋሞ  2. ጎፋ   1. ኦሮሞ 2. አማራ 3. ትግሬ 4. ሌላ (ይገለፅ)_________ |  |
| 507 | የእናት የት/ት ሁኔታ | 1. መደበኛ ት/ት ያልተማረች  2. ማንበብና መፃፍ የማትችል  3.የመጀመሪያ ደረጃ ት/ት የተከታተለች  4. የሁለተኛና ከዛ በላይ ት/ት የተከታተለች |  |
| 508 | የአባት የት/ት ሁኔታ | 1. መደበኛ ት/ት ያልተማረ 2. ማንበብና መፃፍ የማይችል 3. የመጀመሪያ ደረጃ ት/ት የተከታተለ 4. የሁለተኛና ከዛ በላይ ት/ት የተከታተለ |  |
| 509 | አንተን/አንችን ጨምሮ ቤት ውስጥ የሚኖሩ የቤተሰብ አባላት ብዛት ስንት ነው | _____________________ |  |
| 510 | የቤተሰቡ አማካይ ወርሃዊ ገቢ ምን ያህል ነው? |  |  |
| 511 | ከዕርግዝና ጋር ተያያዥነት ያለው የስኳር ህመም አለብሽ ተብለሽ ታውቂያለሽ? (ለሴቶች ብቻ) | 1. አዎ አለ  2. የለም |  |
| 512 | በጊቢ ዉስጥ መጸዳጃ ቤት አለ? | 1. አዎ አለ  2. የለም |  |
| 513 | ቆሻሻ እንዴት ነው የምታስወግዱት | 1. የምናስወግድበት መንገድ የለም  2. ጉድጓድ ውስጥ መክተት  3. ማቃጠል  4. ሜዳ ላይ እንጥላለን  5. ቆሻሻ ቅርጫት ላይ እንጥላለን |  |
| 514 | የመጠጥ ውሃ ከየት ነው የምታገኙት | 1. ከቧንቧ  2. ከተጠበቀ ጉድጓድ/ምንጭ  3. ካልተጠበቀ ጉድጓድ/ምንጭ  4. ከወንዝ |  |
| 515 | አሁን ለ ጠየኩት ልጅ የአልጋ አጎበር ትጠቀሚያለሽ/ማለህ? | 1. አዎ  2. አልጠቀምም |  |
| 516 | ከህፃናት ጤና ወይም አመጋገብ ጋር ተያይዞ ስለህፃናት አያያዝ መረጃ ታገኛለሽ/ህ? | 1. አዎ አገኛለሁ  2. አላገኝም | 601 |
| 517 | ለ518 መልሱ አወ ከሆነ መረጃውን ከየት ንው የምታገኘው/ኝው? | 1. ከጤና ተቋማት  2. ከጤና ኤክስቴንሽን (ከጤና ተቋም ውጭ)  3. ሌላ (ይገለፅ)_________ |  |

**የምግብ ዋስትናን የሚመለከቱ ጥያቄዎች**

| ተ.ቁ | ጥያቄዎች | የመልስ አማራጮች | እለፍ |
| --- | --- | --- | --- |
| 601 | ባለፉት አራት ሳምንታት ዉስጥ በቤተሰቦዎ **በቂ ምግብ የለም** ብለዉ ተጨንቀዋልን? | 1. አይደለም 2. አዎ | 602 |
| 601ሀ | እንዴት ነበር የተከሰተዉ? | 1. በጣም አልፎ አልፎ(1/2 ጊዜ/በ4 ሳምንት 2. አንዳንዴ (ከ 3 እስከ 10 ጊዜ) 3. በተደጋጋሚ (ከ10 ጊዜ በላይ ) |  |
| 602 | ባለፉት አራት ሳምንታት ዉስጥ አቅም ባለመኖር ምክንያት እርሶዎ ወይም ማንኛዉም የቤተሰቦዎ አባል የሆነ **የፈለገዉን ምግብ ለመብላት አልቻለም** ወይ? | 1. አይደለም 2. አዎ | 603 |
| 602ሀ | እንዴት ነበር የተከሰተዉ? | 1. በጣም አልፎአልፎ(1/2ጊዜ /በ 4 ሳምንት  2. አንዳንዴ (ከ 3 እስከ 10 ጊዜ)  3. በተደጋጋም (ከ10 ጊዜ በላይ) |  |
| 603 | ባለፉት አራት ሳምንታት ዉስጥ አቅም ባለመኖር ምክንያት እርሶዎ ወይም ማንኛዉም የቤተሰቦዎ አባል የሆነ **ዉስን የምግብ ዓይነት** በልተዋልን? | 1. አይደለም 2. አዎ | 604 |
| 603ሀ | እንዴት ነበር የተከሰተዉ? | 1. በጣም አልፎ አልፎ (1/2ጊዜ/በ 4 ሳምንት  2. አንዳንዴ (ከ 3 እስከ 10 ጊዜ)  3. በተደጋጋም (ከ1 0 ጊዜ በላይ ) |  |
| 604 | ባለፉት አራት ሳምንታት ዉስጥ ለሎች የምግብ ዓይነቶችን ለማግኘት አቅም ባለመኖሩ እርሶዎ ወይም ማንኛዉም የቤተሰቦዎ አባል የሆነ **ለመብላት የማትፈልጉትን የምግብ ዓይነት** በልታችኋልን? | 1. አይደለም 2. አዎ | 605 |
| 604ሀ | እንዴት ነበር የተከሰተዉ? | 1. በጣም አልፎ አልፎ (1 /2ጊዜ/4 ሳምንት  2. አንዳንዴ (ከ3 እስከ 10 ጊዜ)  3. በተደጋጋም (ከ10 ጊዜበላይ ) |  |
| 605 | ባለፉት አራት ሳምንታት ዉስጥ በቂ ምግብ ባለመኖሩ እርሶዎ ወይም ማንኛዉም የቤተሰቦዎ አባል የሆነ **ከጧዋት ወይም ማታ ከሚገባዉ በታች** በልታችኋልን? | 1. አይደለም 2. አዎ | 606 |
| 605ሀ | እንዴት ነበር የተከሰተዉ? | 1. በጣም አልፎ አልፎ (1 /2ጊዜ/4 ሳምንት  2. አንዳንዴ (ከ3 እስከ 10 ጊዜ)  3. በተደጋጋም (ከ10 ጊዜበላይ ) |  |
| 606 | ባለፉት አራት ሳምንታት ዉስጥ በቂ ምግብ ባለመኖሩ እርሶዎ ወይም ማንኛዉም የቤተሰቦዎ አባል የሆነ **በቀን ከ 3 ጊዜ በታች** በልታችኋልን? | 1. አይደለም 2. አዎ | 607 |
| 606 | እንዴት ነበር የተከሰተዉ? | 1. በጣም አልፎ አልፎ(1ጊዜ/2ጊዜ/4 ሳምንት  2. አንዳንዴ (ከ3 እስከ 10 ጊዜ)  3. በተደጋጋሚ (ከ10 ጊዜበላይ ) |  |
| 607 | ባለፉት አራት ሳምንታት ዉስጥ **ምንም ምግብ በቤተዎ እንዲሁም ምንም ማግኛ አማራጭ** አልነበረምን? | 1. አይደለም 2. አዎ | 608 |
| 607ሀ | እንዴ ትነበር የተከሰተዉ? | 1. በጣም አልፎ አልፎ (1 /2ጊዜ/4 ሳምንት  2. አንዳንዴ (ከ3 እስከ 10 ጊዜ)  3. በተደጋጋሚ (ከ10 ጊዜበላይ ) |  |
| 608 | ባለፉት አራት ሳምንታት ዉስጥ በቂ ምግብ ባለመኖሩ ምክንያት እርሶዎ ወይም የቤተሰቦዎ አባል **እየተራቡ ተኝተዋልን?** | 1. አይደለም 2. አዎ | 609 |
| 608ሀ | እንዴት ነበር የተከሰተዉ? | 1. በጣም አልፎ አልፎ(1ጊዜ/2ጊዜ/4 ሳምንት  2. አንዳንዴ (ከ 3 እስከ 10 ጊዜ)  3. በተደጋጋሚ (ከ10 ጊዜበላይ ) |  |
| 609 | ባለፉት አራት ሳምንታት ዉስጥ በቂ ምግብ ባለመኖሩ ምክንያት ከእርሶዎ ወይም ከቤተሰቦዎ አባል **ሙሉቀንና ማታ ምንም ምግብ ያልበላ ነበርን?** | 1. አይደለም 2. አዎ | ቅጣይ ክፍል |
| 609ሀ | እንዴት ነበር የተከሰተዉ? | 1. በጣም አልፎ አልፎ (1 /2ጊዜ/4 ሳምንት  2. አንዳንዴ (ከ3 እስከ 10 ጊዜ)  3. በተደጋጋሚ (ከ10 ጊዜበላይ) |  |

**አሁን ያለውን የቤተሰብ ሃብት ለማወቅ የሚረዱ ጥያቄዎች (የኢኮኖሚ ደረጃ)**

ከዚህ በታች የተዘረዘሩት እናንተ ቤት መኖራቸውን/አለመኖራቸውን ልትነግረኝ ትችላለህ?

| የንብረት አይነት | ምላሽ | |
| --- | --- | --- |
| **የቤት እንሰሳት** |  |  |
| በሬ | የለም (0) | አለ (1) |
| ላም | የለም (0) | አለ (1) |
| ጥጃ | የለም (0) | አለ (1) |
| በግ | የለም (0) | አለ (1) |
| ፍየል | የለም (0) | አለ (1) |
| ፈረስ | የለም (0) | አለ (1) |
| አህያ | የለም (0) | አለ (1) |
| ዶሮ | የለም (0) | አለ (1) |
| **ቋሚ ንብረቶች** |  |  |
| ቴሌቪዥን | የለም (0) | አለ (1) |
| ሬዲዮ | የለም (0) | አለ (1) |
| የኤሌክትሪክ አቅርቦት | የለም (0) | አለ (1) |
| ማቀዝቀዣ | የለም (0) | አለ (1) |
| መደበኛ ስልክ | የለም (0) | አለ (1) |
| ተንቀሳቃሽ ስልክ | የለም (0) | አለ (1) |
| መኪና | የለም (0) | አለ (1) |
| ሞተር ብስክሌት | የለም (0) | አለ (1) |
| ብስክሌት | የለም (0) | አለ (1) |
| ጋሪ | የለም (0) | አለ (1) |
| ወርቅ፣ ገንዘብ | የለም (0) | አለ (1) |
| የምትኖሩበት ቤት የናንተ ነው? | አይደለም (0) | ነው (1) |
| የእርሻ መሬት አላችሁ | የለንም (0) | አለን (1) |
| **የማምረቻ ግብዓቶች** |  |  |
| ማረሻ | የለም (0) | አለ (1) |
| ምሳር | የለም (0) | አለ (1) |
| መኮትኮቻ | የለም (0) | አለ (1) |
| አካፋ | የለም (0) | አለ (1) |
| ማጭድ | የለም (0) | አለ (1) |
| የንብ ቀፎ | የለም (0) | አለ (1) |
| **የቤት ሁኔታ** |  |  |
| ቤት ውስጥ ቧንቧ አለ | የለም (0) | አለ (1) |
| የቤቱ ወለል ምን አይነት ነው | አፈር/በእንሰሳት እዳሪ የተለቀለቀ (0) | ሲሚንቶ/እንጨት(1) |
| አልጋ | የለም (0) | አለ (1) |
| ጠረጴዛ | የለም (0) | አለ (1) |
| ወንበር | የለም (0) | አለ (1) |
| የኤሌክትሪክ ማብሰያ (ስቶቭ) | የለም (0) | አለ (1) |

ላደረጉልኝ ተሳትፎ አመሰግናለሁ፡፡
